# Motivational profiles of medical students: Association with study effort, academic performance and exhaustion

**DOI:** 10.1186/1472-6920-13-87

**Published:** 2013-06-19

**Authors:** Rashmi A Kusurkar, Gerda Croiset, Francisca Galindo-Garré, Olle Ten Cate

**Affiliations:** 1 VUmc School of Medical Sciences, Postal address: VU University Medical Center Amsterdam, Institute of Education and Training, Postbus 7057, A-114, Amsterdam, MB 1007, the Netherlands; 2 Department of Biostatistics, VU University Medical Center Amsterdam, Amsterdam, the Netherlands; 3 Center for Research and Development of Education, UMC Utrecht, Postal address: Center for Research and Development of Education, University Medical Center Utrecht, P.O. Box # 85500, Utrecht, GA 3508, the Netherlands

**Keywords:** Motivation, SDT, Learning outcomes, Academic performance, Intrinsic motivation, Controlled motivation, Motivational profiles

## Abstract

**Background:**

Students enter the medical study with internally generated motives like genuine interest (intrinsic motivation) and/or externally generated motives like parental pressure or desire for status or prestige (controlled motivation). According to Self-determination theory (SDT), students could differ in their study effort, academic performance and adjustment to the study depending on the endorsement of intrinsic motivation versus controlled motivation. The objectives of this study were to generate motivational profiles of medical students using combinations of high or low intrinsic and controlled motivation and test whether different motivational profiles are associated with different study outcomes.

**Methods:**

Participating students (N = 844) from University Medical Center Utrecht, the Netherlands, were classified to different subgroups through K-means cluster analysis using intrinsic and controlled motivation scores. Cluster membership was used as an independent variable to assess differences in study strategies, self-study hours, academic performance and exhaustion from study.

**Results:**

Four clusters were obtained: High Intrinsic High Controlled (HIHC), Low Intrinsic High Controlled (LIHC), High Intrinsic Low Controlled (HILC), and Low Intrinsic Low Controlled (LILC). HIHC profile, including the students who are interest + status motivated, constituted 25.2% of the population (N = 213). HILC profile, including interest-motivated students, constituted 26.1% of the population (N = 220). LIHC profile, including status-motivated students, constituted 31.8% of the population (N = 268). LILC profile, including students who have a low-motivation and are neither interest nor status motivated, constituted 16.9% of the population (N = 143). Interest-motivated students (HILC) had significantly more deep study strategy (p < 0.001) and self-study hours (p < 0.05), higher GPAs (p < 0.001) and lower exhaustion (p < 0.001) than status-motivated (LIHC) and low-motivation (LILC) students.

**Conclusions:**

The interest-motivated profile of medical students (HILC) is associated with good study hours, deep study strategy, good academic performance and low exhaustion from study. The interest + status motivated profile (HIHC) was also found to be associated with a good learning profile, except that students with this profile showed higher surface strategy. Low-motivation (LILC) and status-motivated profiles (LIHC) were associated with the least desirable learning behaviours.

## Background

Students enter the medical study with different types of motives. These could be generated internally, like interest in biology or in helping people or desire for intellectual challenge, and/or from external factors, like desire for monetary rewards, prestige or pressure from parents [1-9]. According to Self-determination Theory (SDT), intrinsic motivation is seen when an activity is done out of genuine interest, and controlled motivation is seen when an activity is done because of external factors. The former would classify as “intrinsic motivation” and the latter would classify as “controlled motivation” [10,11]. These types of motivation endorsed by the students are considered important in predicting how students adjust to their study, how much effort they are willing to invest in their study, performance in medical school and preference of specialty [11,12]. It has been found that intrinsic motivation, as compared to controlled motivation, leads to greater creativity [13], less superficial information processing [14], more deep learning [15,16], higher achievement [17,18], enhanced well-being or adjustment [19,20], decreased drop-out intention and behaviour [21,22].

Most studies of motivation in medical education have explored the relationship between motivation and study outcomes as group variables [9], which is called a variable-oriented approach [23]. This approach is useful for understanding how motivation influences academic achievement and also the direction of influence. Another approach is to look at how individual students differ in their motivation and how this relates to their academic outcomes. This person-oriented approach builds on the variable-oriented approach and helps to understand the motivational orientation of individual students.

SDT posits that every individual has combinations of intrinsic and controlled motivation for every activity, either one of the two being dominant or both being equally dominant. This creates subgroups among students which can be studied through a person-oriented approach [23]. In this paper, we particularly address the motivation of students for studying medicine. It can be hypothesized that study outcomes would be different among students depending on which subgroup he or she belonged to. For example, intrinsically motivated students are likely to exhibit a different type of study behaviour resulting in more study hours and deep learning strategy as compared to students with controlled motivation who more often show a surface learning strategy [10,24,25].

The present study was carried out to test the hypothesis - derived from SDT [23] - that different subgroups of medical students, made on the basis of the combination of their intrinsic and controlled motivation, are related to differences in learning outcomes and academic performance. We refer to these combinations or patterns of motivation types within subgroups as “motivational profiles”. We aimed to answer two questions through this study:

What types of motivational profiles, combining intrinsic and controlled motivation, exist among medical students?

Are differences in motivational profiles, based on the above combinations, associated with differences in study effort, study strategy, academic performance and exhaustion from study?

We expected to find four motivational profiles namely High Intrinsic High Controlled (HIHC), High Intrinsic Low Controlled (HILC), Low Intrinsic High Controlled (LIHC) and Low Intrinsic Low Controlled (LILC), in line with a study carried out by Vansteenkiste et al.^a^[1] on secondary school and college students [23].

We have put forth some speculations based on the literature in following sentences in order to make it easier for the readers to understand these profiles in practice. The different motivation profiles of students mentioned below have been actually found in studies in medical education [1-9], though these examples have never been attributed to motivational profiles before. HIHC profile would be seen in a student who endorsed both intrinsic and controlled motivation in high quantity, e.g. a student who was interested in medicine, but also driven by the prestige of the profession. HILC profile would be seen in a student who endorsed intrinsic motivation in high quantity and controlled motivation in low quantity, e.g. a student studying medicine only because of interest in patients or biology. LIHC profile would be seen in a student who was following the study only for monetary rewards or parental pressure. LILC profile would be seen in a student who was indifferent to the choice of medicine, performed well in a qualifying exam, had the chance to enter medicine and decided to try it. In the present study, the profiles described above have been labelled according to the items of the scales used for measurement of motivation and what they would mean to the readers, in order to understand the practical relevance of these profiles (See Table 1). HIHC has been labelled as “Interest + status motivated” profile, HILC as “Interest-motivated” profile, LIHC as “Status-motivated” profile and LILC as “Low-motivation” profile.

**Table 1:** Motivational profiles based on SDT

	**High CM**	**Low CM**
High IM	**Interest + status motivated**	**Interest-motivated**
	**(HIHC)**	**(HILC)**
Low IM	**Status-motivated**	**Low-motivation**
	**(LIHC)**	**(LILC)**

*IM* Intrinsic Motivation*, CM* Controlled Motivation.

Based on SDT [10,24] and the research done in general education on motivational profiles [23,26], we hypothesized that:

•The interest-motivated profile (HILC) would be associated with a deep learning strategy and more hours of study, better academic performance and low exhaustion.

•The interest + status motivated profile (HIHC) would be associated with a surface learning strategy, good academic performance and higher exhaustion.

•The status-motivated (LIHC) and low-motivation (LILC) profiles would be associated with surface learning strategy, fewer hours of study, lower academic performance and high exhaustion.

## Methods

### Sample

Students from all six years of the medical course at University Medical Center Utrecht, the Netherlands, were included in this study. The first part of the study was carried out over a period of three months towards the end of 2009. The internet based data collection programme “Survey Monkey – http://www.surveymonkey.com/” was used to send out an electronic questionnaire to two thousand and twenty students. The second part of the study was carried out in October 2010 which included collecting academic performance data from one term, i.e. six months of the academic year 2009–2010. The data were anonymized before carrying out the analyses.

### Ethical approval

Medical education research in The Netherlands was exempt from ethical approval requirement when we carried out this study. To make sure that we complied with the rules laid down by the Declaration of Helsinki the students were explained that the participation in the study was voluntary, there was guarantee of confidentiality and anonymity and that non-participation would not cause them any harm. They could also choose to withdraw from the study at any time without giving any reason. Written informed consent was obtained from all the participants.

### Instruments used

An internet-based electronic survey which contained some personal proforma questions, the Academic Motivation Scale (AMS) [27] to measure intrinsic and controlled motivation, a question on number of self-study hours per week, Study Process Questionnaire (SPQ) [28] to assess the study strategies (deep and surface) of the students and “exhaustion from study” scale from Maslach Burnout Inventory-Student Survey (MBI-SS) [29] was used. We modified the AMS, which been originally designed for college and university students, for use in medical students [30] and investigated its validity and reliability. Intrinsic motivation scores were calculated from the AMS as an average of the scores on the three subscales of intrinsic motivation [27]. Controlled motivation scores were calculated by taking an average of introjected regulation and external regulation extrinsic motivation scores as described in SDT literature [14,15]. We did not use the amotivation subscale of AMS as it was not included in the theoretical basis and hypotheses of our study. According to SPQ, deep study strategy scores reflected use of study strategy by students to create an in-depth understanding of the study material, whereas surface study strategy scores reflected the use of study strategy to memorize facts from the study material without deep understanding [28]. The SPQ also has good validity and reliability in medical students [12]. We collected the academic performance results in terms of ECs (European Credits) credits and GPA (Grade Point Average) attained by the students in one term, September 2009 up to February 2010. ECs are awarded after completing a course and passing the exam on that course and GPA is the weighted average of grades (weighted according to the ECs that can be obtained) attained by the students.

### Statistical analyses

The validity and reliability of all the questionnaires used was investigated through confirmatory factor analysis and calculation of Cronbach’s alpha value for each subscale used.

The data were analyzed using SPSS version 15.0. Students were clustered into different motivational profiles using K-means clustering (using squared Euclidean distances and iterative method) on the Z-scores of their intrinsic and controlled motivation. The variable “intrinsic motivation” meant the scores on items in AMS inquiring about interest in the medical subject matter. The variable “controlled motivation” meant the scores on items in AMS inquiring about the need for status or prestige or money as a reason to study medicine.

The variances in intrinsic and controlled motivation scores explained by the cluster solution were calculated using analysis of variance ANOVA. For the cluster solution to be acceptable, it needed to explain a minimum of 50% variance in the intrinsic and controlled motivation scores. We carried out a double-split cross-validation procedure as described by Vansteenkiste et al. to examine the stability of the cluster solution [23]. Using cluster membership as an independent variable, we compared study strategies, self-study hours, exhaustion from study, ECs and GPAs using multivariate analysis of covariance (MANCOVA) method.

## Results

There was a response rate of 42% since 849 students out of 2020 filled out the survey. Out of these 73.2% were females and 26.8% were males. This was a representative sample as the Cox d effect size of the difference between gender distribution of the study and actual (69.6% females and 30.4% males) student population was 0.1, which is considered a small effect size [31]. Some students did not fill out some of the scales. We carried out the analyses of the learning variables and outcomes with the respective completed responses. The internal consistencies of the different scales used in the survey were acceptable, Cronbach’s alpha values of all being above 0.70. First we computed correlations between all independent and dependent variables which are given in Table 2. We found that intrinsic motivation was significantly positively correlated with deep strategy towards study (r = 0.46), self-study hours (r = 0.095) and GPA (r = 0.108) and significantly negatively correlated with surface strategy towards study (r = −0.152) and exhaustion from study (r = −0.179). In contrast, controlled motivation was significantly negatively correlated with self-study hours (r = −0.115) and GPA (r = −0.117) and significantly positively correlated with surface strategy (r = 0.260) and exhaustion from study (r = 0.088). These correlations are in line with those found in the SDT literature [14,15].

**Table 2:** Correlations between all variables measured and differences between males and females

**Variable**	**Males mean (SD)**	**Females mean (SD)**	**T test (p value)**	**1**	**2**	**3**	**4**	**5**	**6**	**7**	**8**
**1 Intrinsic motivation**	4.82 (0.72)	4.91 (0.73)	−1.47 (0.14)	-							
**2 Controlled motivation**	4.23 (1.14)	3.86 (1.18)	4.09 (0.00***)	0.332**	-						
**3 Deep strategy**	2.83 (0.61)	2.79 (0.64)	0.84 (0.397)	0.460**	0.050	-					
**4 Surface strategy**	2.39 (0.59)	2.34 (0.58)	0.991 (0.322)	−0.152**	0.260**	−0.239**	-				
**5 Self-study hours/week**	12.92 (7.12)	13.97 (7.10)	−1.84 (0.065)	0.095**	−0.115**	0.332**	−0.153**	-			
**6 European credits**	19.95 (7.86)	20.92 (8.40)	−1.446 (0.149)	0.008	0.066	−0.017	0.017	−0.068	-		
**7 GPA**	7.22 (0.98)	7.45 (0.84)	−3.00 (0.003**)	0.108**	−0.117**	0.195**	−0.250**	−0.032	−0.032	-	
**8 Exhaustion from study**	1.88 (1.07)	2.07 (1.02)	−2.38 (0.017*)	−0.179**	0.088*	−0.123**	−0.290**	0.085*	−0.052	−0.151**	-

*p < 0.05, ** p < 0.01, ***p < 0.001, GPA-Graded Point Average.

All the scores obtained from the students were converted into Z-scores in order to make them comparable. The Z-scores on intrinsic motivation and controlled motivation were used to cluster the students into different motivational profiles.

**Figure 1:**
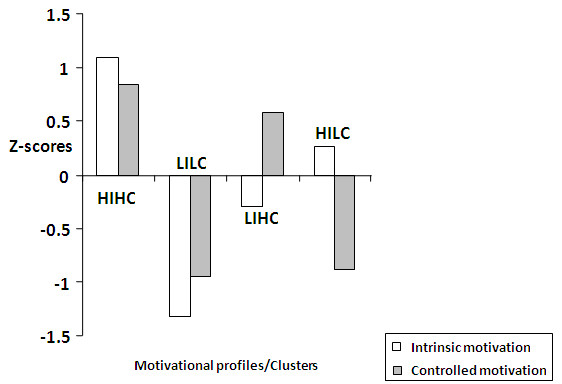
**Motivational profiles through cluster analysis.***HIHC: Interest + Status motivated profile, LILC: Low-motivation profile, LIHC: Status-motivated profile, HILC: Interest-motivated profile.*

Five outliers were removed from the data as cluster analyses are highly sensitive to outliers. For the 844 students included in the analyses, we tried fitting 3-cluster, 4-cluster and 5-cluster solutions according to the methods described for cluster analyses. Based on the theory and the explained incremental variance a 4-cluster solution, as we had anticipated, fitted the data best. It explained 69.7% variance in the intrinsic motivation scores and 64.4% variance in the controlled motivation scores. The 4 clusters obtained are shown in Table 3 and Figure 1.

**Table 3:** Distribution of students among the 4 clusters/profiles along with the gender distribution

**Cluster membership**	**Interest + status motivated (HIHC)**	**Interest- motivated (HILC)**	**Status-motivated (LIHC)**	**Low-motivation (LILC)**	**Total**
**Intrinsic motivation Z-score – Mean (SD)**	5.703 (0.419)	5.071 (0.370)	4.639 (0.392)	3.857 (0.452)	4.887 (0.734)
**Controlled motivation Z- score – Mean (SD)**	4.960 (0.742)	2.903 (0.699)	4.640 (0.574)	2.823 (0.881)	3.960 (1.187)
**No. of students in cluster (%)**	213 (25.2%)	220 (26.1%)	268 (31.8%)	143 (16.9%)	844 (100%)
**Males**	61 (27%)	35 (15.5%)	92 (40.7%)	38 (16.8%)	226 (100%)
**Females**	152 (24.6%)	185 (30%)	176 (28.4%)	105 (17%)	618 (100%)
**Chi-square statistic for gender**	21.42				
**Significance**	p = 0.00				

We empirically validated the results of our cluster analyses by double-split cross-validation and found that both random samples yielded similar cluster solutions.

The distribution of males and females in the different clusters was significantly different with a Chi square statistic of 21.42 and p < 0.001 (see Table 3). The status-motivated (LIHC) profile had the highest percentage of male students (40.7%) and the interest-motivated (HILC) profile had the highest percentage of female students (30%) and the lowest percentage of male students (15.5%). The students from different years of the curriculum (1 to 6) were well-distributed among the different profiles, therefore we did not control for year of curriculum while performing the analyses.

Since we found significantly different distribution of gender between the profiles and significant differences in some learning variables (Table 2), we decided to analyze the differences between learning variables and outcomes between the clusters after correcting for gender differences. After performing the checks required to test the assumptions of MANCOVA, we conducted a MANCOVA using cluster membership as an independent variable, learning variables and outcomes as dependent variables and gender as a covariate (Table 4).

For the learning variables and outcomes, the Wilk’s lambda was significant, F = 68.674, P < 0.001, partial eta squared = 0.508 i.e. a large effect size [32], meaning that the learning variables and outcomes were significantly different for different profiles. Gender showed a multivariate effect, F = 4.366, p < 0.001, partial eta squared = 0.062 i.e. a medium effect [32], meaning that there were significant differences because of gender (Table 4).

**Table 4:** Differences between learning variables and outcomes among different motivational profiles [MANCOVA]

	**Interest + status motivated (HIHC) Mean (SD)**	**Interest-motivated (HILC) Mean (SD)**	**Status-motivated (LIHC) Mean (SD)**	**Low-motivation (LILC) Mean (SD)**	**F**	**Eta squared (% of variance explained)**
**Intrinsic motivation (n = 844, scale score = 1-7)**	5.70_a_ (0.41)	5.07_b_ (0.36)	4.63_c_ (0.39)	3.85_d_ (0.45)	452.72***	0.694 (69.4%)
**Controlled motivation (n = 844, scale score = 1-7)**	4.96_a_ (0.74)	2.90_b_ (0.69)	4.64_c_ (0.57)	2.82_b_ (0.88)	352.77***	0.639 (63.9%)
**Deep strategy (n = 709, scale score = 1-5)**	3.10_a_ (0.55)	2.93_b_ (0.62)	2.64_c_ (0.58)	2.40_d_ (0.65)	31.64 ***	0.137 (13.7%)
**Surface strategy (n = 709, scale score = 1-5)**	2.39_a_ (0.62)	2.14_b_ (0.52)	2.50_a_ (0.57)	2.37_a_ (0.59)	11.48 ***	0.054 (5.4%)
**Self -study hours (n = 796)**	14.16_a,b_ (7.67)	14.65_a_ (7.69)	12.77_b_ (6.31)	13.20_a,b_ (6.61)	3.05**	0.015 (1.5%)
**ECs (n = 780)**	21.39_a_ (8.15)	19.84_a_ (8.77)	20.95_a_ (8.07)	20.23_a_ (7.96)	1.126	0.004 (0.4%)
**GPA (n = 780)**	7.41_a_ (0.93)	7.62_a,b_ (0.76)	7.20_c_ (0.93)	7.35_a,c_ (0.86)	5.78***	0.028 (2.8%)
**Exhaustion from study (n = 844, scale score = 0-6)**	1.91_a_ (1.03)	1.83_a,b_ (0.99)	2.14_a,c_ (1.03)	2.29_c_ (1.06)	5.04**	0.025 (2.5%)

**p < 0.05, ***p < 0.001.

The means with different subscripts are significantly different from each other, i.e. a mean with subscript “a” is significantly different from a mean with subscript “b” or “c”.

Effect sizes from Eta squared: Small = 0.01-0.06, Medium = 0.06-0.138, Large > 0.138 [32].

The interest-motivated students had significantly more deep strategy and significantly less surface strategy towards study as compared to the status-motivated (p < 0.001) and low-motivation (p < 0.001) students. This was as we had expected to find. The interest + status motivated students had significantly more deep (p = 0.01) and surface strategies (p < 0.001) as compared to the interest-motivated students. It probably means that the interest + status motivated students employ both deep and surface strategies as and when required. The interest-motivated students had significantly less exhaustion from study as compared to the low-motivation (p < 0.001) and the status-motivated (p = 0.009) students. The interest + status motivated students also had significantly less exhaustion from study as compared to low-motivation students (p = 0.01). The interest-motivated students showed significantly more self-study hours as compared to status-motivated students (p = 0.003) and also more self-study hours than interest + status motivated and low-motivation students, but this difference did not reach statistical significance. There was no difference between the ECs obtained by the different profiles. The interest-motivated students had the highest GPAs, which were significantly higher than the low-motivation (p = 0.021) and status-motivated (p < 0.001) students, but the difference did not reach statistical significance in comparison with interest + status motivated profile. The effect sizes of all the dependent variables ranged from small to moderate.

## Discussion

In a previous study we have analyzed the relationship between motivation, learning and academic performance using structural equation modelling for a variable-oriented approach [12]. We found motivation to have a positive effect on academic performance mediated by study effort and strategy [12]. In the present person-oriented study we find that different motivational profiles of students exist when combining high and low intrinsic and high and low controlled motivation in every individual through analysis of subgroups [23,26]. The present study builds on our previous study by providing an insight into the motivational forces working in every individual in relation to the different study outcomes. Understanding these different combinations could also help in customizing mentoring and support activities for the different groups of students. Motivational profiles type of analysis yields complementary information to studying intrinsic and controlled motivation as group variables.

To our knowledge, this is the first study in medical education which classifies students according to their motivational profiles. Creating subgroups using cluster analysis of the motivational variable has been done before in medical education [33], but all possible combinations of intrinsic or controlled motivation and how these affect learning in medicine has not been studied. Our study also carries the work of Vansteenkiste et al. a step further as it utilizes actual academic performance results rather than self-reported performance results [23].

In this study, we found that males had significantly higher controlled motivation as compared to females, which has been found in earlier studies [26,33], but we found no difference in intrinsic motivation. When we looked into the distribution of genders within the profiles, we found that males were represented more in the status-motivated profile and females were represented more in the interest-motivated profile. Vansteenkiste et al. found similar distribution among the clusters in their study [23]. We have consistently found differences in motivation between males and females in our other studies [12,34,35]. We have also found before that males need to invest more time in self-study in order to get GPAs which are comparable to females [12]. The implication of these findings would be that males who have higher controlled motivation need different type of mentoring than females.

Though Vansteenkiste et al. hypothesized that interest-motivated students would perform better than interest + status motivated students, engage in more meaningful study and have better well-being than interest + status motivated students, they actually found that these students were indeed significantly better on performance and test anxiety, but not on the other learning parameters [23]. Ratelle et al. used quality, i.e. intrinsic and controlled motivation and amotivation scores, and quantity, i.e. high, moderate and low motivation to create different profiles [26]. They could not find evidence for all the profiles which they had hypothesized about. They found that the interest + status motivated students performed as well as the interest-motivated students, but the interest-motivated students were more persistent in their study [26].

We found that the interest-motivated students had the optimal learning profile with high deep strategy, low surface strategy, more time spent in self-study, good ECs, high GPAs and low exhaustion from study. Both low-motivation and status-motivated profiles had the least desirable learning characteristics with satisfactory ECs, but lower deep strategy, higher surface strategy, fewer hours in self-study, lower GPA and higher exhaustion from study. These findings are in line with the study by Vansteenkiste et al. [23] Interest + status motivated students scored surprisingly high on deep study strategy and low on exhaustion from study [23]. On all the parameters they did as well as the interest-motivated students, except on the GPA, where the difference was not statistically significant. This was the difference we found from Vansteenkiste et al. study in which GPA (self-reported grades) of interest-motivated students was significantly higher [23]. We also found interest + status motivated profile similar to interest-motivated profile on exhaustion from study. The interest + status motivated students in our study showed high deep strategy and good GPA probably because their intrinsic motivation scores were higher than their controlled motivation scores, even though the controlled motivation scores, themselves were quite high. We probably did not find differences in ECs because these credits are awarded on completion of a course and passing the exam on that course, independent of how high a student may score in the exam. Thus ECs may not be discriminative enough among different students. Since Ratelle et al. found interest + status profile to have higher dropout behaviour as compared to status-motivated profile [26], dropout behaviour could be added in any further studies.

It would be worthwhile to investigate whether different motivational profiles would benefit by different ways of monitoring and mentoring during their medical study. It would also be of interest to find out whether these motivational profiles remain stable during medical study or change according to the learning environment and experience. We would recommend a longitudinal study design to study this aspect of profiling. Another area of interest for further research would be to investigate whether different motivational profiles show differences in effectiveness and attitudes towards the practice of medicine in their professional life.

### Limitations

We would like to highlight one important limitation of this study. We would have liked to use the variable *autonomous motivation* which is calculated as an average of intrinsic motivation and identified regulation (another subscale of AMS) in this study [10,24]. AMS has been used earlier in studies on college and university students, but not on students in professional education [21,26,27]. The items of this subscale are such that most students in professional education would answer positively. Thus, this subscale is not likely to discriminate in our study population [30]. For this reason we carried out all further analyses with *intrinsic motivation* as the clustering variable. This is, in principle, justified as intrinsic motivation is a prototype of autonomous motivation, but it may have reduced the sensitivity of the variable (motivation) to pick out differences between the groups. To be able to overcome this difficulty in further studies we would recommend development of an identified regulation scale specifically for students of health professions. Though the sample size for all variables was enough to find differences, the fact that all students did not fill out all questionnaires is a limitation of this study This study has been carried out in only one university and hence the findings have limited generalizability. Our findings in regard to the relationship of motivational profiles with study outcomes need to be confirmed by further research on other populations. This line of research needs further development in other universities, preferably in other countries, because of differences in gender distribution in medical education.

## Conclusions

High Intrinsic Low Controlled motivation (interest-motivated profile) is associated with good study hours, deep learning strategy, good academic performance and low exhaustion from study. High Intrinsic High Controlled motivation (interest + status motivated profile) was also found to be associated with a good learning profile, except that students with this profile showed high surface strategy. Low Intrinsic High Controlled (status-motivated profile) and Low Intrinsic Low Controlled (low-motivation profile) motivation were associated with the least desirable learning behaviours.

## Endnote

^a^Vansteenkiste et al. 2009 and Ratelle et al. 2007 used autonomous motivation scores instead of intrinsic motivation scores for creating subgroups. Autonomous motivation means motivation which an individual perceives as originating from within his self. Intrinsic motivation is the prototype of autonomous motivation. We have used intrinsic motivation to describe both these studies throughout the paper for ease of understanding.

## Abbreviations

IM: Intrinsic motivation; CM: Controlled motivation; HIHC: High intrinsic high controlled; HILC: High intrinsic low controlled; LIHC: Low intrinsic high controlled; LILC: Low intrinsic low controlled; ECs: European credits; GPA: Grade point average

## Competing interests

The authors declare that they have no competing interests.

## Authors’ contributions

RAK, GC and TJC contributed to designing the study. RAK collected the data and analysed it together with FGG. All authors contributed to the interpretation of results and discussion. All authors contributed to important intellectual content of the paper and approved the final version of the manuscript.

## Authors’ information

RA Kusurkar, MD, PhD is an Assistant Professor and the Head of Research in Medical Education, VUmc School of Medical Sciences, Amsterdam, the Netherlands. This work was carried out when RAK was pursuing her PhD in Medical Education at the Center for Research and Development of Education, UMC Utrecht, The Netherlands. G Croiset, MD, PhD is Professor of Medical Education and Director of the Undergraduate and Graduate Programme in Medicine, VUmc School of Medical Sciences, Amsterdam, the Netherlands. F Galindo-Garré, PhD, is a Statistician in the Department of Biostatistics, VUmc Amsterdam, the Netherlands. ThJ ten Cate, PhD, is Professor of Medical Education and Director of the Center for Research and Development of Education, UMC Utrecht, the Netherlands.

## Pre-publication history

The pre-publication history for this paper can be accessed here:

http://www.biomedcentral.com/1472-6920/13/87/prepub
